# Therapeutic targets and pharmacological mechanisms of *Coptidis Rhizoma* against ulcerative colitis: Findings of system pharmacology and bioinformatics analysis

**DOI:** 10.3389/fphar.2022.1037856

**Published:** 2022-11-30

**Authors:** Yuanming Yang, Yiwei Hua, Weihuan Chen, Huan Zheng, Haomeng Wu, Shumin Qin, Shaogang Huang

**Affiliations:** ^1^ Dongguan Hospital of Guangzhou University of Chinese Medicine, Dongguan, Guangdong, China; ^2^ The Second Clinical College of Guangzhou University of Chinese Medicine, Guangzhou, China; ^3^ State Key Laboratory of Dampness Syndrome of Chinese Medicine, The Second Affiliated Hospital of Guangzhou University of Chinese Medicine, Guangdong Provincial Hospital of Chinese Medicine, Guangzhou, China; ^4^ Guangdong-Hong Kong-Macau Joint Lab on Chinese Medicine and Immune Disease Research, Guangzhou, China; ^5^ Guangdong Provincial Key Laboratory of Clinical Research on Traditional Chinese Medicine Syndrome, Guangzhou, China; ^6^ Yang Chunbo Academic Experience Inheritance Studio of Guangdong Provincial Hospital of Chinese Medicine, Guangzhou, China

**Keywords:** *Coptidis Rhizoma*, Huanglian, ulcerative colitis, system pharmacology, bioinformatics analysis

## Abstract

Evidence of the advantages of *Coptidis Rhizoma* (CR) for the treatment of ulcerative colitis (UC) is accumulating. However, research revealing the targets and molecular mechanisms of CR against UC is scarce. In this research, a bioinformatics analysis was performed to carry out the physicochemical properties and biological activities of phytochemicals in CR and analyze the binding activities, targets, biological functions and mechanisms of CR against UC. This research shows that the CR’s key phytochemicals, which are named Coptisine, Berberrubine, Berlambine, Berberine, Epiberberine, Obacunone, Worenine, Quercetin, (R)-Canadine, Magnograndiolide, Palmatine and Moupinamide, have ideal physicochemical properties and bioactivity. A total of 1,904 potential phytochemical targets and 17,995 UC-related targets are identified, and we finally acquire 233 intersection targets between key phytochemicals and disease. A protein-protein interaction network of 233 common targets was constructed; and six hub targets were acquired with a degree greater than or equal to median, namely TP53, HSP90AA1, STAT3, ESR1, MYC, and RELA. The enrichment analysis suggested that the core targets may exert an impact on anti-inflammatory, immunoregulatory, anti-oxidant and anti-fibrosis functions mainly through the PI3K/ART signaling pathway, Th17 differentiation signaling pathway, inflammatory bowel disease signaling pathway, etcetera. Also, a molecular docking analysis shows that the key phytochemicals have strong affinity for binding to the core targets. Finally, the interaction network of CR, phytochemicals, targets, GO functions, KEGG pathways and UC is constructed. This study indicates that the key phytochemicals in CR have superior drug likeness and bioactivity, and the molecular mechanism of key phytochemicals against UC may be *via* the signaling pathway mentioned above. The potential and critical pharmacological mechanisms provide a direction for future research.

## Highlights

1. The physicochemical properties and bioactivity of key phytochemicals in *Coptidis Rhizoma* were assessed. The results show that the key phytochemicals, which are named Coptisine, Berberrubine, Berlambine, Berberine, Epiberberine, Obacunone, Worenine, Quercetin, (R)-Canadine, Magnograndiolide, Palmatine and Moupinamide, have ideal drug likeness and biological activity.
2. All biotargets of the key phytochemicals in CR against Ulcerative Colitis are identified, and core targets, namely TP53, HSP90AA1, STAT3, ESR1, MYC, and RELA, are recognized. Molecular docking indicated that the key phytochemicals have strong affinity for binding to the core targets.
3. The key phytochemicals in *Coptidis Rhizoma* have anti-inflammatory, immunoregulatory, anti-oxidant and anti-fibrosis effects by targeting core targets *via* the PI3K-ART signaling pathway, Th17 differentiation signaling pathway and inflammatory bowel disease signaling pathway.

## Introduction

Ulcerative Colitis (UC) is a type of inflammatory bowel disease characterized by mucosal inflammation initiating in the rectum and extending continuously towards the colon with the manifestation of abdominal pain, tenesmus and bloody diarrhea (Magro et al., 2017; Kobayashi et al., 2020), which severely impairs patients’ quality of life, physical and mental health (Lin et al., 2022). The pathophysiology of UC is multifaceted and partially understood, but it is thought to be influenced by environment, genetic factors, immune dysregulation and gut microbiome changes (Kobayashi et al., 2020). Current treatments for UC include 5-aminosalicylic acid formulations, glucocorticoids, immunosuppressants, and biologics. An increasing amount of evidence shows that long-term use of these medicines may have potentially serious side effects, such as bone marrow and liver toxicity, pancreatitis, an increased risk of non-melanoma skin cancer, and lymphoma (Magro et al., 2017). Therefore, it is imperative to develop an alternative, safe and effective therapy for treating UC.

As a natural medicine, *Coptidis Rhizoma* (CR) has been used to alleviate the notable symptoms of UC for thousands of years in China, such as bloody diarrhea, tenesmus and abdominal pain (Wang et al., 2020; Lu et al., 2021; Niu et al., 2021). Previous studies have demonstrated that CR has the pharmacological effects of anti-inflammatory, anti-bacterial, anti-oxidant, anti-tumor and other pharmacological effects (Nagajyothi et al., 2014; Kwon et al., 2021; Kang et al., 2022). However, the mechanism by which different bioactive components target UC has not been scientifically and systematically investigated.

**FIGURE 1:**
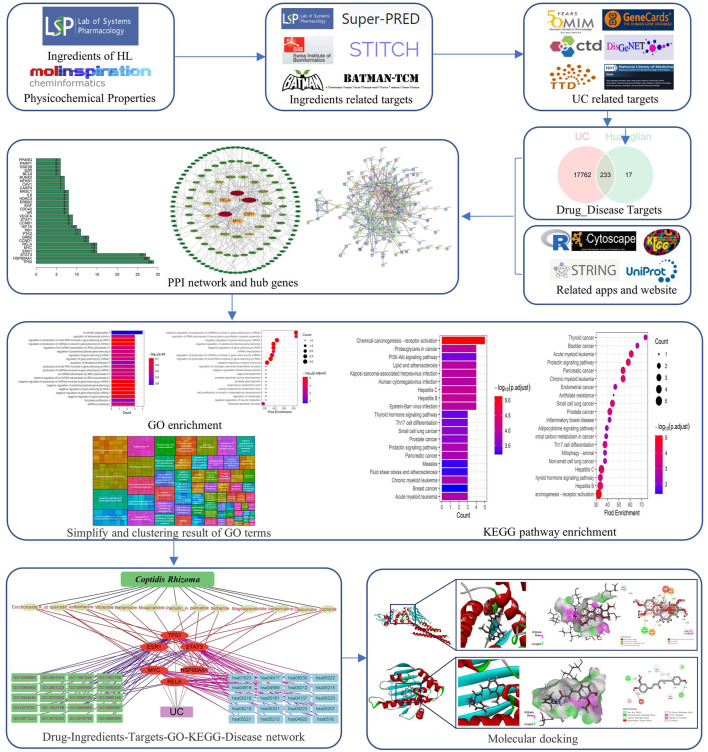
The flow diagram of this research.

Network pharmacology is an emerging bioinformatics approach used to evaluate the pharmacological effects of natural medicines based on their multi-component, multi-targets and multi-pathway action patterns (Yuan et al., 2017; Li et al., 2021). The purpose of the present work was to elucidate the detailed mechanism behind the activity of CR against UC with the methods of systematic pharmacology and bioinformation analysis. Moreover, molecular docking was also used to confirm the interaction mechanism between the core target and the key component. In this way, the present study may provide the fundamental instructions for further research on the mechanism of CR and its active compounds for treating UC. The flow sketch of our research is depicted in Figure 1.

## Materials and methods

### Collection of *Coptidis Rhizoma* active ingredients

The Traditional Chinese Medicine Systems Pharmacology Database and Analysis Platform (TCMSP, https://tcmsp-e.com/tcmsp.php, accessed on 25 June 2022) was employed to obtain phytochemicals of CR. Adjusting the pharmacokinetics index so that the oral bioavailability (OB) was greater than or equal to 30% (Xu et al., 2012) and the drug-like (DL) index was greater than or equal to 0.18 (Jia et al., 2020) was another way to narrow down the list of active ingredients.

### Analysis of physicochemical properties and biological activities

The Molinspiration server (https://www.molinspiration.com, accessed on 25 June 2022) was used to acquire the molecular descriptors, drug likeness and bioactivity (Xie et al., 2021). Lipinski’s Rule of Five (Lipinski et al., 2001), consisting of the following parameters: logP, molecular weight (MW), number of hydrogen bond acceptors (n-ON) and number of hydrogen bond donors (n-OHNH), was used to evaluate the pharmacokinetic properties of the identified compounds. The biological bioactivity scores for each of the ingredients, including G-protein coupled receptor (GPCR) ligand, ion channel modulator, kinase inhibitor, nuclear receptor ligand, protease inhibitor and enzyme inhibitor, were calculated (Rashid, 2020). The following formula was used to calculate an additional percentage of absorption (%ABS) value: %ABS = 109—[0.345*TPSA] (Xie et al., 2021).

### Collection of *Coptidis Rhizoma* related targets

Pharmacological targets for each of active ingredients were identified and collected from online databases, such as TCMSP (https://tcmsp-e.com/tcmsp.php, accessed on 25 June 2022), Swiss Target Prediction (http://www.swisstargetprediction.ch, accessed on 25 June 2022), A Bioinformatics Analysis Tool for Molecular Mechanism of Traditional Chinese Medicine (BATMAN-TCM, http://bionet.ncpsb.org.cn/batman-tcm, accessed on 25 June 2022), Search Tool for Interacting Chemicals (STITCH, http://stitch.embl.de, accessed on 25 June 2022) and Super-PRED (https://prediction.charite.de/subpages/target_prediction.php, accessed on 25 June 2022). All candidate targets were duplicated after standard gene symbol transformation with review and human setting in the Uniprot database (Li et al., 2021).

### Collection of ulcerative colitis related targets

UC-related targets were gathered from six open-source databases listed as follows: GeneCards (https://www.genecards.org, accessed on 25 June 2022), Online Mendelian Inheritance in Man (OMIM, https://www.omim.org, accessed on 25 June 2022), Comparative Toxicogenomics Database (CTD, http://ctdbase.org, accessed on 25 June 2022), DisGeNET (https://www.disgenet.org, accessed on 25 June 2022), NCBI gene (https://www.ncbi.nlm.nih.gov, accessed on 25 June 2022), Therapeutic Target Database (TTD, http://db.idrblab.net/web, accessed on 25 June 2022). All the targets of disease were identified after eliminating duplicates. The R-language package ggvenn (https://rdocumentation.org/packages/ggvenn) was used to discover intersecting targets between the potential protein targets of active ingredients in CR and UC. The intersection was the final target for CR against UC.

### Analysis of protein-protein interaction network and hub targets

The STRING database (https://string-db.org, accessed on 26 June 2022) was employed to construct the PPI network with the minimum required interaction score of 0.95 and a species restriction of “*Homo sapiens*.” The PPI network was then visualized and analyzed using Cytoscape software (version 3.9.1, Boston, MA, United States, accessed on 26 June 2022). The Network Analyzer plugin of Cytoscape was utilized to analyze the topology parameters, including the minimum and maximum degrees of freedom in the network. The hub targets were selected based on degree values, with the upper limit of the filtering range set to the greatest degree-value and the lower limit taken as the median degree of freedom in the topological data (Li et al., 2021).

### Assays of functional process and molecular pathways

Enrichment analysis of Gene Ontology (GO) function and Kyoto Encyclopedia of Genes and Genomes (KEGG) pathway of the core targets was performed and visualized through R-language packages, including “ClusterProfiler” (version 4.2.2), “org.Hs.eg.DB” (version 3.14.0) and “ggplot2” (version 3.3.6). The species was limited to “*Homo sapiens*,” and any term with an adjusted *p*-value less than 0.05 was deemed significantly enriched. The GO terms and KEGG pathways were illustrated in the results ranked by count or fold enrichment. Since enrichment analysis may produce a long list of significant terms with a lot of duplicate information (Gu and Hübschmann 2022), the R-language package, “rrvgo” (version 1.6.0) (Sayols, 2020), was employed to simplify enrichment results.

### Molecular docking verification of ingredients and targets

Molecular docking analysis was performed to investigate the binding affinity between the key phytochemicals and the core targets. The 3D structure of each of the compounds was obtained from the PubChem database (https://pubchem.ncbi.nlm.nih.gov, accessed on 28 June 2022). The crystal structure of the hub target was retrieved from the Protein Data Bank database (PDB, https://www.rcsb.org, accessed on 28 June 2022). BIOVIA Discovery Studio 2019 was used to extract ligands and water molecules from the crystal structure complex, as well as for protein preparation and docking grid creation. The first docking site in receptor cavities generated by BIOVIA Discovery Studio 2019 was selected as the binding site, and the Lib Dock method was adopted to perform the molecular docking. To verify the reliability of our docking result, AutoDock Vina was also employed to perform molecular docking between receptor and ligand.

## Results

### Physicochemical properties and bioactivity of active compounds

A total of 48 phytochemicals of CR were retrieved from the TCMSP, and 14 active phytochemicals were obtained on the basis of OB ≥ 30% and DL ≥ 0.18, the basic information of this phytochemicals is shown in Table 1. The physicochemical properties of each compound were evaluated in our study. Twelve active phytochemicals (except for Palmidin A and Corchoroside A_qt from a total of 14 active phytochemicals) met Lipinski’s rule of five with no violations. The twelve compounds with a value of TPSA < 140 Å^2^ and %ABS > 63% were in the range of ideal oral bioavailability as shown in Table 2. A molecule with a bioactivity score greater than 0.00 is anticipated to exhibit substantial biological activity, while those with a score of between −5.0 and 0.00 are predicted to be moderately active, and it is presumed to be inactive for the score below −5.0 (Rashid, 2020). The physiological role of the ingredients in CR may be associated with a variety of mechanisms, including possible interactions with GPCR ligands, ion channel modulators, kinase inhibitors, nuclear receptor ligands, protease inhibitors and enzyme inhibitors. Coptisine exhibited promising enzyme inhibitor and ion channel modulator affinities with bioactivity scores greater than or equal to 0.8 > 0, moderate GPCR ligand, kinase inhibitor, protease inhibitor and nuclear receptor ligand affinities with bioactivities values between −5.0 and 0. The results indicated that Coptisine, as well as Berberine, have superior enzyme inhibitor and ion channel modulator affinity (enzyme inhibitor > ion channel modulator > GPCR ligand > kinase inhibitor > protease inhibitor > nuclear receptor ligand). More of the detailed information about other compounds is summarized in Table 3.

**TABLE 1:** Basic information of key phytochemicals of CR.

Mol ID	Compounds name	Molecular formular	OB (%)	DL	Structure
MOL001458	Coptisine	C19H14NO4^+^	30.67	0.86	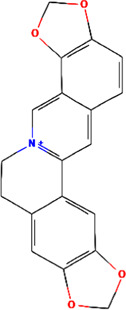
MOL000762	Palmidin A	C30H22O8	35.36	0.65	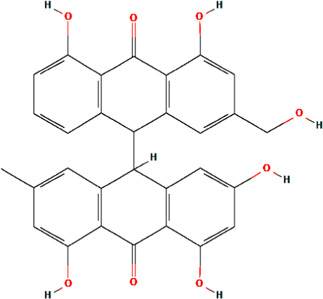
MOL002894	Berberrubine	C19H16ClNO4	35.74	0.73	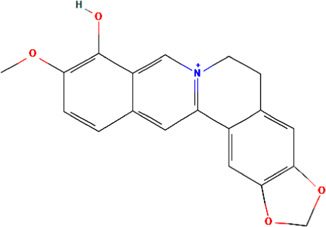
MOL002904	Berlambine	C20H17NO5	36.68	0.82	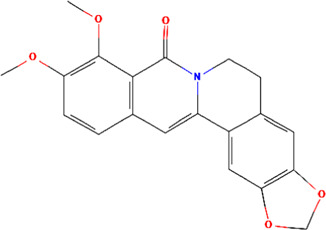
MOL001454	Berberine	C20H18NO4^+^	36.86	0.78	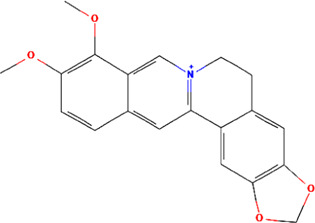
MOL002897	Epiberberine	C20H18NO4^+^	43.09	0.78	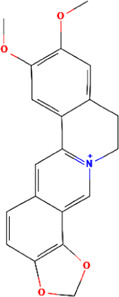
MOL013352	Obacunone	C26H30O7	43.29	0.77	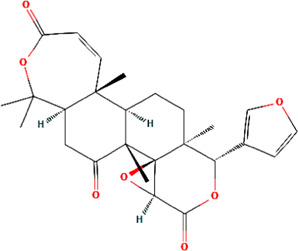
MOL002668	Worenine	C20H16NO4^+^	45.83	0.87	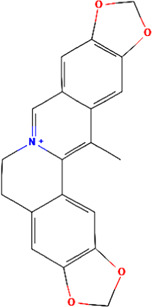
MOL000098	Quercetin	C15H10O7	46.43	0.28	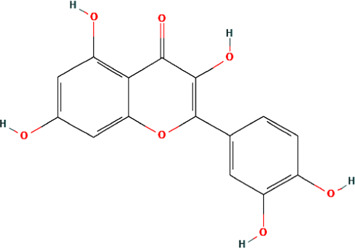
MOL002903	(R)-Canadine	C20H21NO4	55.37	0.77	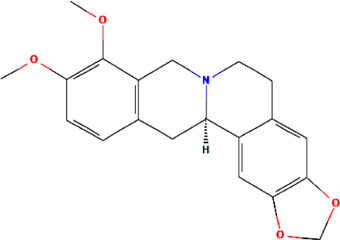
MOL000622	Magnograndiolide	C15H22O4	63.71	0.19	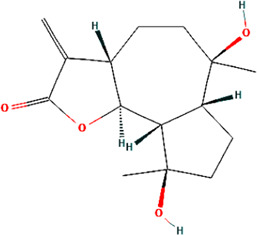
MOL000785	Palmatine	C21H22NO4^+^	64.6	0.65	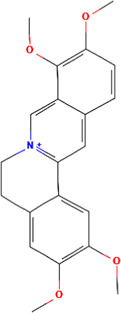
MOL008647	Moupinamide	C18H19NO4	86.71	0.26	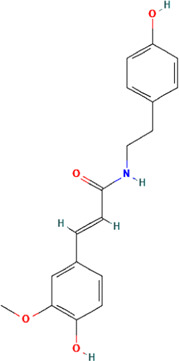
MOL002907	Corchoroside A_qt	C29H42O9	104.95	0.78	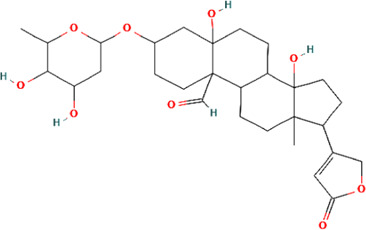

**TABLE 2:** Physicochemical properties of key phytochemicals of CR.

Compound standard criteria	%ABS	MiLogP < 5	TPSA (Å^2^)	n-atoms	MW < 500	n-ON < 10	n-OHNH < 5	n-violations ≤ 1	n-rotb ≤ 10	MV
Coptisine	94.91	0.24	40.82	24	320.32	5	0	0	0	269.14
Palmidin A	55.34	4.81	155.51	38	510.50	8	6	2	2	429.68
Berberrubine	91.12	−0.08	51.81	24	322.34	5	1	0	1	278.77
Berlambine	88.66	3.13	58.94	26	351.36	6	0	0	2	301.58
Berberine	94.91	0.20	40.82	25	336.37	5	0	0	2	296.30
Epiberberine	94.91	−0.00	40.82	25	336.37	5	0	0	2	296.30
Obacunone	76.10	3.80	95.35	33	454.52	7	0	0	1	405.76
Worenine	94.91	0.62	40.82	25	334.25	5	0	0	0	285.70
Quercetin	63.68	1.68	131.35	22	302.24	7	5	0	1	240.08
(R)-Canadine	95.14	2.99	40.17	25	339.39	5	0	0	2	305.61
Magnograndiolide	85.97	0.72	66.76	19	266.34	4	2	0	0	252.49
Palmatine	94.91	−0.05	40.82	26	352.41	5	0	0	4	323.46
Moupinamide	81.81	4.53	78.79	23	313.35	5	3	0	6	289.44
Corchoroside A_qt	59.74	0.80	142.76	38	534.64	9	4	1	4	490.57

**TABLE 3:** Bioactivity scores of key phytochemicals of CR.

Compound	GPCR ligand	Ion channel modulator	Kinase inhibitor	Nuclear receptor ligand	Protease inhibitor	Enzyme inhibitor
Coptisine	−0.06	0.80	−0.22	−0.82	−0.33	0.89
Palmidin A	0.07	−0.11	0.08	0.26	−0.01	0.17
Berberrubine	−0.12	0.66	−0.24	−0.80	−0.44	0.87
Berlambine	0.13	−0.16	0.07	−0.28	−0.10	0.03
Berberine	−0.11	0.71	−0.27	−0.78	−0.35	0.82
Epiberberine	−0.10	0.71	−0.23	−0.86	−0.38	0.81
Obacunone	0.15	−0.05	−0.57	0.60	0.07	0.41
Worenine	−0.04	0.64	−0.32	−0.07	−0.35	0.52
Quercetin	−0.06	−0.19	0.28	0.36	−0.25	0.28
(R)-Canadine	0.19	−0.05	−0.43	−0.48	−0.26	-0.06
Magnograndiolide	0.14	0.02	−0.40	1.01	−0.14	0.71
Palmatine	−0.10	0.74	−0.22	−0.65	−0.29	0.81
Moupinamide	0.10	−0.06	−0.16	0.05	−0.05	0.02
Corchoroside A_qt	0.10	−0.03	−0.36	0.31	0.13	0.78

Bioactivity score of >0 represented promising activity, bioactivity score between −5.0 and 0.00 represented moderate activity, and bioactivity score of <−5.0 represented no activity.

### Target identification of *Coptidis Rhizoma* and ulcerative colitis

**FIGURE 2:**
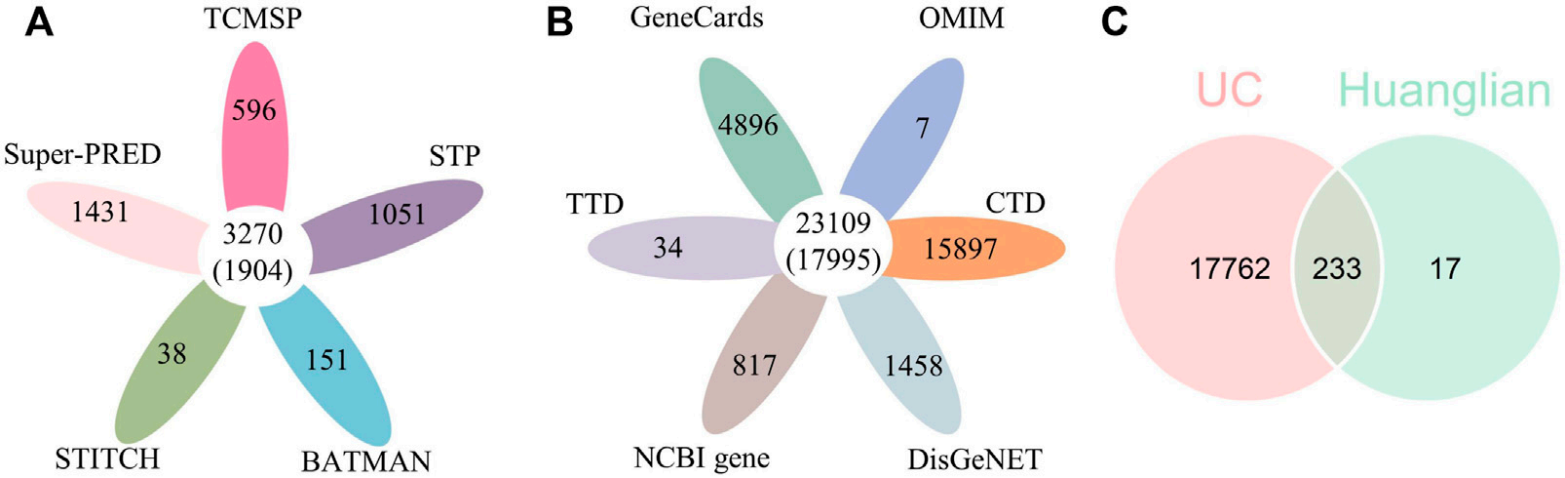
**(A)** The number of target genes related to ingredients of CR from five open-source databases. **(B)** The number of target genes related to UC from six open-source databases. **(C)** Venn diagram depicting common target genes between UC and CR (Huanglian).

Five open-source databases, including TCMSP (596), Swiss Target Prediction (1051), BATMAN-TCM (151), STITCH (38) and Super-PRED (1431), were used to obtain CR-related targets. The CR-related target set was established by combining a union of the screened results, 1,904 targets were obtained after the removal of duplications and transferring gene symbols (Figure 2A). UC-related targets were gathered from six open-source databases: GeneCards (4896), OMIM (7), CTD (15897), DisGeNET (1458), NCBI gene (817), and TTD (34). 17,995 targets were acquired after checking for duplications (Figure 2B). Finally, 233 common target genes between CR and UC were identified (Figure 2C). The 233 target genes were further used to construct the PPI network of CR against UC, and the hub genes were screened for enrichment and pathway analysis.

### PPI network and hub targets

**FIGURE 3:**
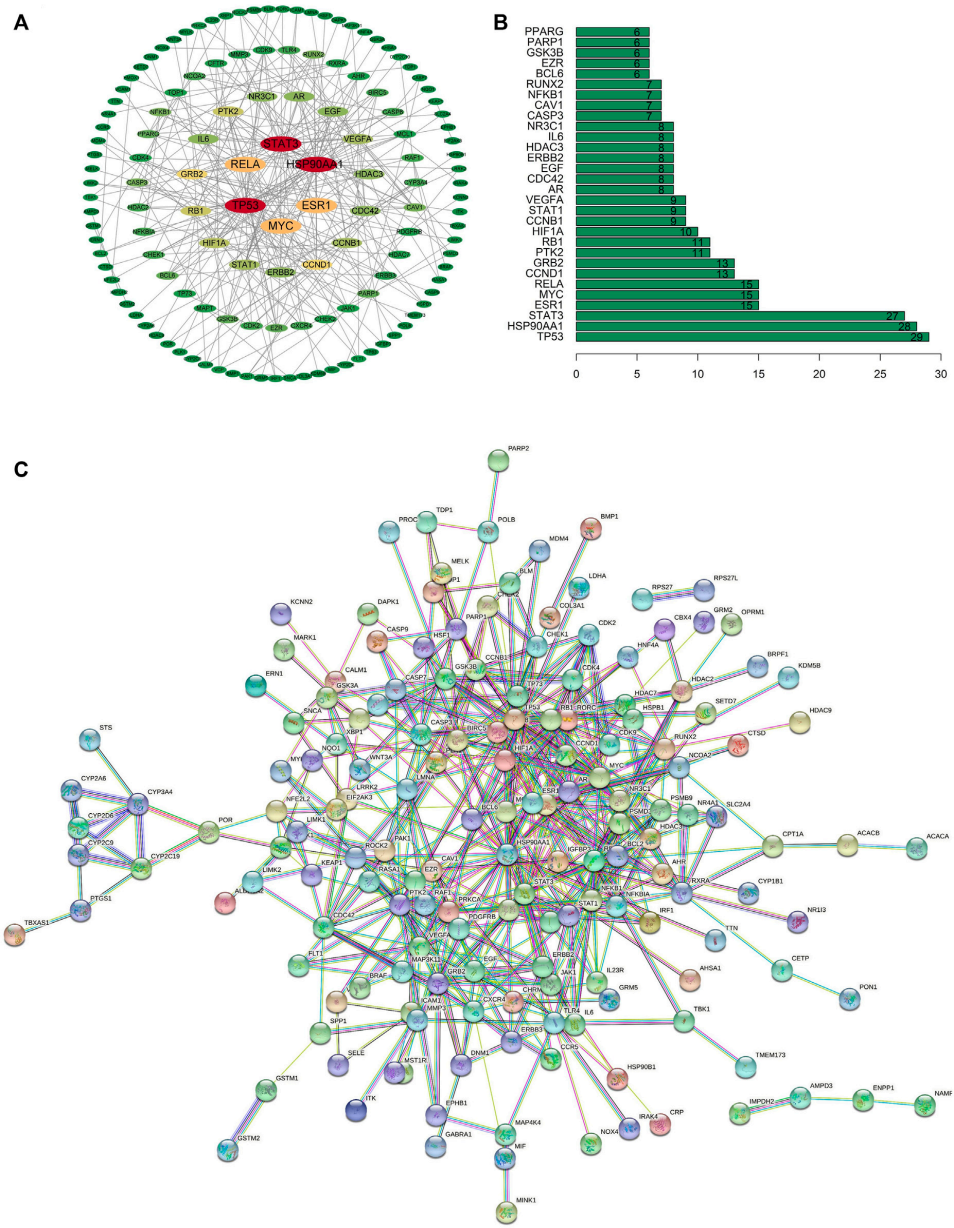
**(A)** PPI network visualized with Cytoscape, the color of each nodes represented degree of freedom. **(B)** Top 30 of targets were ranked from a histogram. **(C)** PPI network exported from STRING database.

The PPI network was constructed by importing the 233 intersecting targets to the STRING database with the minimum required interaction score of 0.95 and species limited to “*Homo sapiens*.” The PPI network was visualized by Cytoscape as shown in Figure 3A. Cytoscape’s Network Analyzer plugin was used to calculate the topological properties of the PPI network. The minimum degree of freedom of the target is 1, the maximum degree is 29 and the medium degree of freedom is 15. The top 30 degrees of freedom were illustrated in Figure 3B. The hub targets were acquired with the inclusion criterion of the degree of freedom greater than or equal to medium 15. Six core genes were screened with the inclusion criterion. Their names were cellular tumor antigen p53 (TP53), heat shock protein HSP 90-alpha (HSP90AA1), signal transducer and activator of transcription-3 (STAT3), estrogen receptor (ESR1), myc proto-oncogene protein (MYC) and transcription factor p65 (RELA) (Figures 3A–C).

### Gene Ontology BPs enrichment and kyoto encyclopedia of genes and genomes pathway analysis of core targets

**FIGURE 4:**
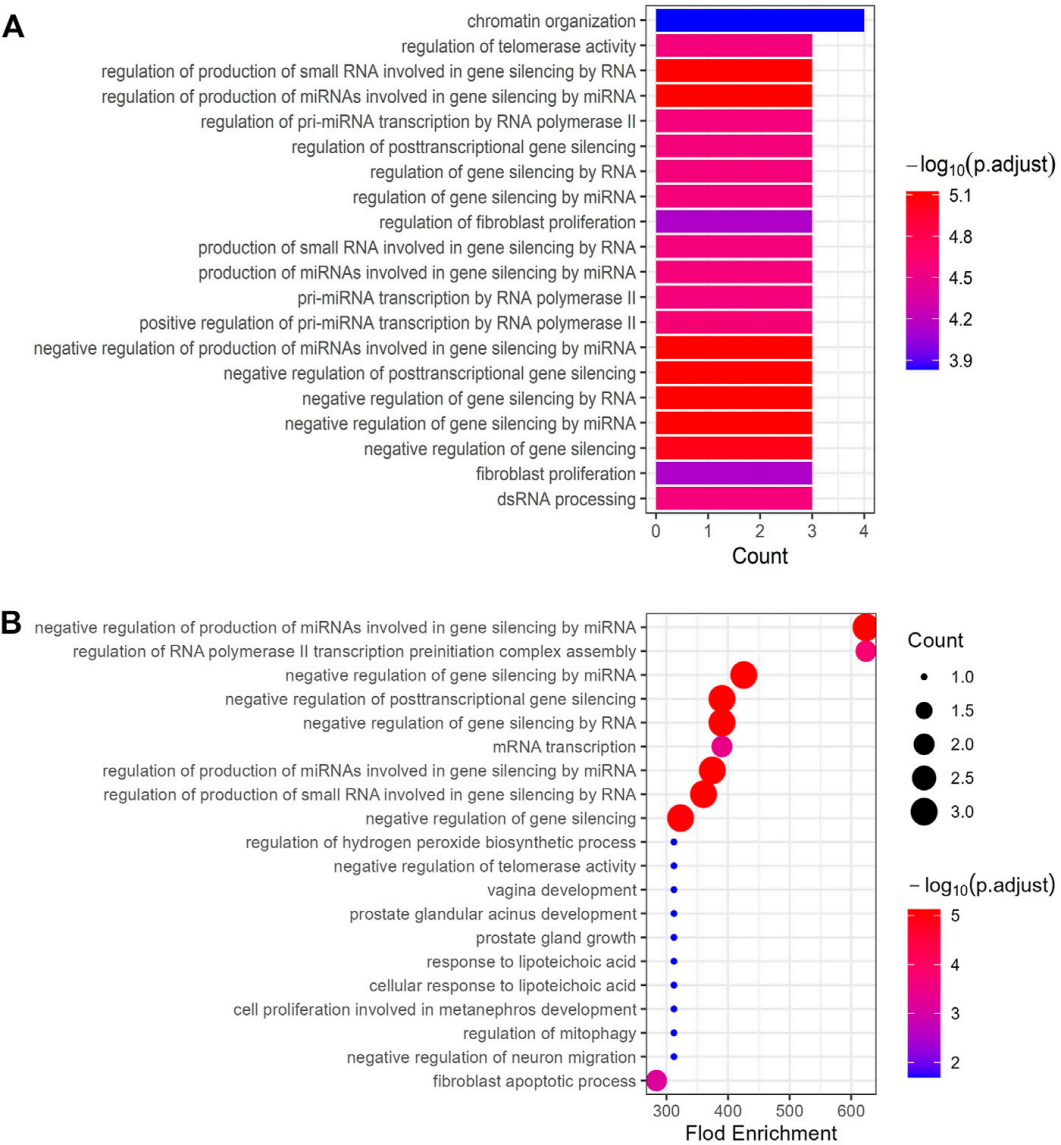
**(A)** The bar plot shows the hub genes enriched top 20 BP items ranked by counts. **(B)** The bubble diagram shows the hub genes enriched top 20 BP items ranked by fold enrichment.

**FIGURE 5:**
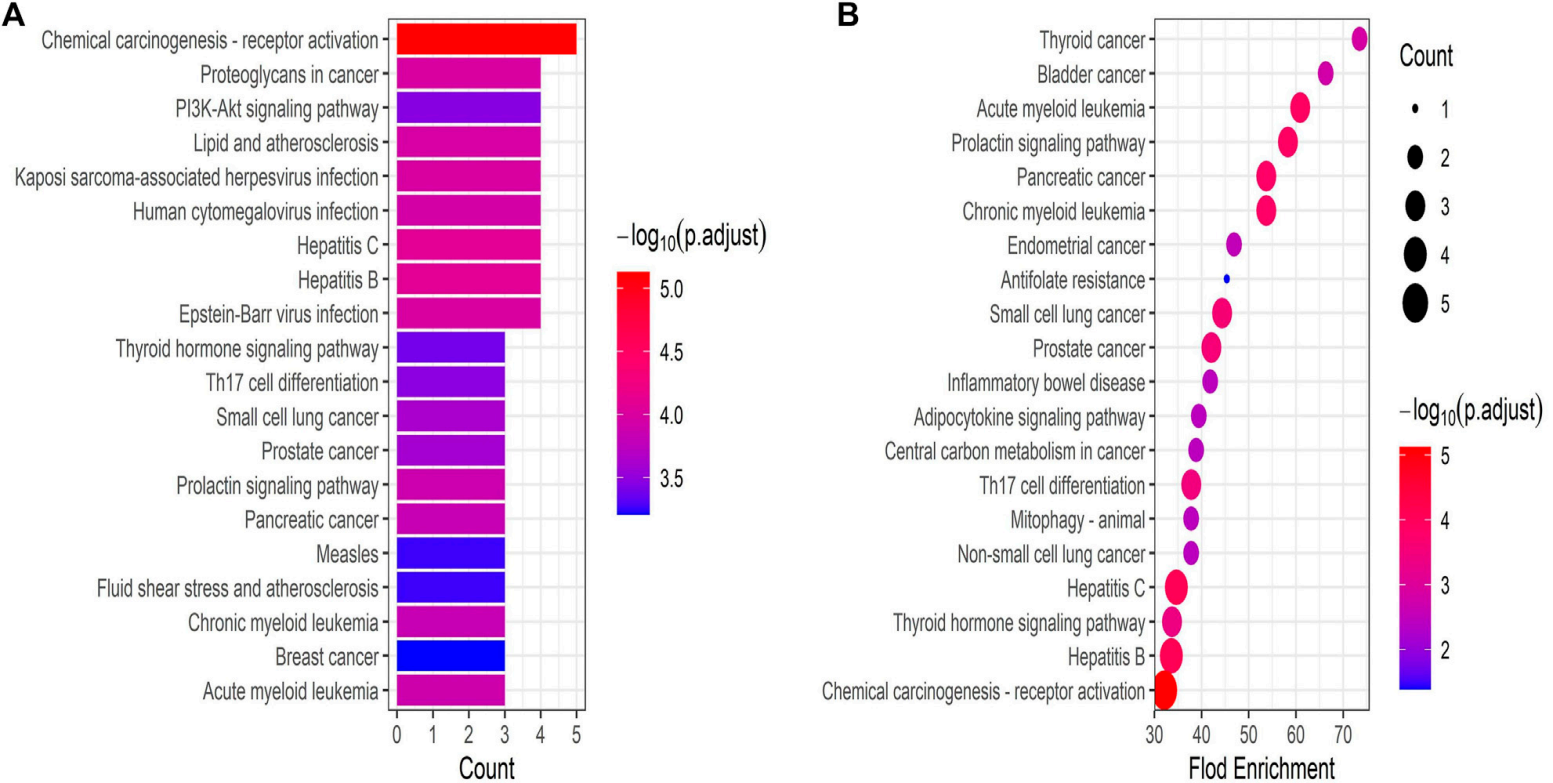
**(A)** The bar plot shows the hub genes enriched top 20 KEGG enrichment pathways ranked by counts. **(B)** The bubble diagram shows the hub genes enriched top 20 KEGG enrichment pathways ranked by fold enrichment.

**FIGURE 6:**
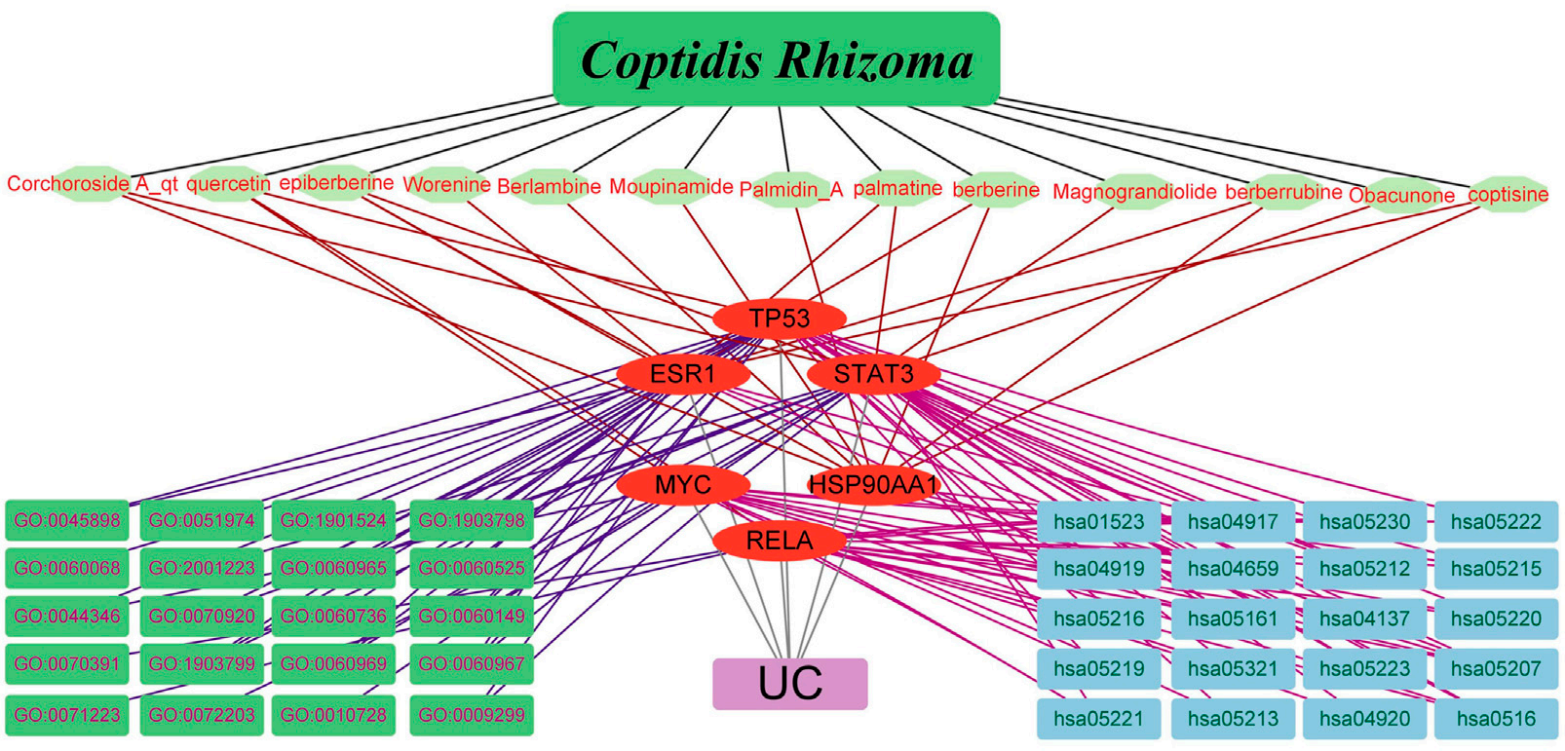
Network visualization from bioinformatics analysis highlighting the detailed interactions of CR-ingredient-target-GO-KEGG-UC.

R-language packages were used to analyze the GO biological process enrichment and KEGG pathway of the six core targets. A total of 623 GO terms for biological processes were obtained, the top 20 count and the top 20 fold enrichment results were visualized using a histogram and a bubble diagram (Figures 4A,B). Considering that enrichment analysis may include highly redundant information, simplified enrichment results performed by the R-package were displayed in Supplementary Figure S1. Biological process enrichment mainly contained the cellular response to interleukin-6, fibroblast apoptotic process, negative regulation of gene silencing by RNA, positive regulation of pro-miRNA transcription by RNA polymerase II, response to UV, fibroblast proliferation, T cell activation involved in immune response, positive regulation of interleukin-8 production, regulation of telomerase activity, and so on. In KEGG analysis, a total of 61 KEGG pathway of the core targets were acquired. The top 20 count and the top 20 fold enrichment results were displayed using a histogram and a bubble chart (Figures 5A,B). The KEGG pathways associated with UC were mainly involved in the PI3K-ART signaling pathways, Th17 cell differentiation, inflammatory bowel disease, adipocytokine signaling pathway, et cetera. The enriched GO functions and KEGG pathways of CR against UC were strongly associated with inflammation, immune response, cell cycle processes and lipid metabolism. Finally, the interaction network composed of CR, phytochemicals, targets, GO functions, KEGG pathways and UC was constructed to reveal the therapeutic target and pharmacological mechanism of CR against UC (Figure 6).

### Binding activities of the key phytochemicals to core targets

**FIGURE 7:**
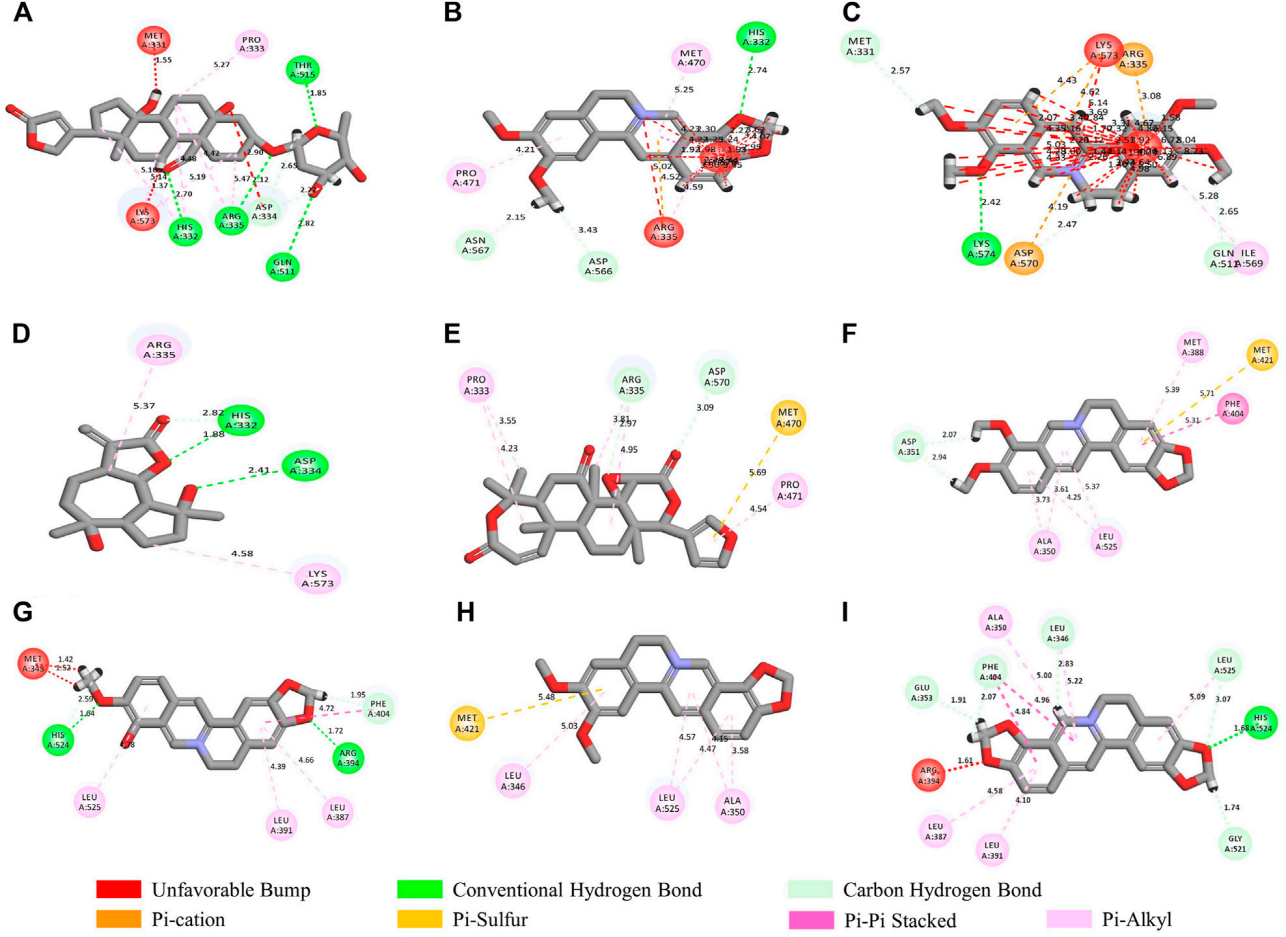
**(A)** Corchoroside A_qt and STAT3; **(B)** Epiberberine and STAT3; **(C)** Palmatine and STAT3; **(D)** Magnograndiolide and STAT3; **(E)** Obacunoneand STAT3; **(F)** Berberine and ESR1; **(G)** Berberrubine and ESR1; **(H)** Epiberberine and ESR1; **(I)** Coptisine and ESR1.

Molecular docking was performed to determine the possible binding of the key phytochemicals in CR with the core targets of UC. In our research, two different molecular docking softwares were used to enhance the reliability of docking results. The lib docking score analyzed by Discovery Studio and the binding energy generated by AutoDock Vina are summarized in Table 4. The docked complexes showed the binding affinities and interactions between ligand and receptor are shown in Figure 7. The greater lib dock score and the lower binding energy of the phytochemicals binding to the target, the greater affinity between the phytochemicals and the targets. The molecular docking results suggested that the key compounds of CR could bind with TP53, HSP90AA1, STAT3, ESR1, MYC, and RELA. Among the key phytochemicals, Quercetin could bind with TP53 (PDB ID, 1A1U), MYC (PDB ID, 1A93), and RELA (PDB ID, 1NFI) with relatively higher lib dock scores and lower binding energy respectively. Within the compounds bound to HSP90AA1 (PDB ID, 1BYQ), Corchoroside A_qt presented excellent binding affinity with the highest lib dock score of 140.99 compared with others. Similar results were also found in the docking results of STAT3 (PDB ID, 6NJS), Corchoroside A_qt displayed the most significant binding affinity with STAT3 with the lowest binding energy of −12.6 kcal/mol. The result of lib dock score and binding energy also shows that the key compounds have better binding affinity with ESR1 (PDB ID, 1A52).

**TABLE 4:** Molecular docking of core targets and key phytochemicals.

Targets	Phytochemicals	Lib Dock score	Binding energy (kcal/mol)
TP53 (1A1U)	Quercetin	82.52	−6.0
HSP90AA1 (1BYQ)	Berlambine	98.87	−6.7
	Moupinamide	110.81	−6.0
	Palmidin_A	123.22	−7.0
	Berberine	104.02	−6.6
	Berberrubine	106.54	−6.6
	Coptisine	109.62	−6.8
	Corchoroside A_qt	140.99	−10.6
	Epiberberine	113.56	−6.4
MYC (1A93)	Quercetin	95.94	−3.1
RELA (1NFI)	Quercetin	127.95	−6.1
STAT3 (6NJS)	Corchoroside A_qt	131.03	−12.6
	Epiberberine	93.26	−7.5
	Palmatine	85.72	−7.0
	Magnograndiolide	84.42	−7.1
	Obacunone	106.78	−7.9
ESR1 (1A52)	Berberine	79.54	−6.9
	Berberrubine	92.32	−6.8
	Epiberberine	96.41	−6.4
	Palmatine	89.62	−6.6
	Coptisine	94.41	−6.6
	Worenine	83.3	−6.4

## Discussion

With increasing prevalence, UC affects millions of individuals worldwide, increasing the risk of colon cancer (Barberio et al., 2022). Considering the adverse effects of first-line treatment, it is urgent to find safe and effective alternative medicines. Numerous studies have demonstrated that CR can ameliorate UC and its complications (Niu et al., 2021; Xie et al., 2022; Yang Y et al., 2022). However, the therapeutic mechanism of CR exerted on UC has not been scientifically and systematically elucidated. In the present study, the biotargets, functional processes and molecular pathways of CR against UC are identified and depicted using systematic pharmacology and bioanalysis techniques. The findings demonstrate that phytochemicals from CR possess promising drug likeness and biological activities and have great potential to become a beneficial therapeutic approach for UC.

Firstly, we investigated the phytochemicals that meet the criteria of OB ≥ 30% and DL ≥ 0.18, which were regarded as the active components. A total of 14 ingredients were found in CR, 13 of the active ingredients potentially target the core genes of UC. The herb (CR), phytochemicals, targets, GO functions, KEGG pathways and disease (UC) interaction network demonstrated that the core targets of UC are modulated by multiple compounds in a manner of multi-targeted modulation, and the core targets exert multiple GO functions and KEGG pathways against UC. Moreover, the physicochemical properties and bioactivity of the active phytochemicals of CR, including drug likeness, absorption, distribution, metabolism and excretion (ADME), were evaluated according to Lipinski’s Rule of Five, TPSA and %ABS (Rashid, 2020). Except for Palmidin A and Corchoroside A_qt, the majority of the active compounds fit with the simplified Lipinski’s Rule of Five, and the values of TPSA < 140 Å^2^, %ABS > 63.68% fell within the range of ideal oral bioavailability. The results demonstrate that these active phytochemicals have no restrictions in terms of drug likeness or ADME. Furthermore, a molecular with a bioactivity score greater than 0 has a significant likelihood of exerting great bioactivity (Xie et al., 2021). Bioactivity investigation revealed that the pharmacological effect of active phytochemicals in CR involves multiple mechanisms, including GPCR ligands, ion channel modulators, kinase inhibitors, nuclear receptor ligands, protease inhibitors and enzyme inhibitors. Hence, we could speculate that the key active phytochemicals in CR have a synergistic effect in the treatment of UC.

In the bioinformatics analysis, a total of 233 potential targets of CR against UC were obtained, and six targets with degree values greater than or equal to the median of 15 were selected as the hub targets in the PPI network. The six hub genes are named TP53, HSP90AA1, STAT3, ESR1, MYC, and RELA. TP53 is the master regulator of apoptosis and senescence (Rabellino et al., 2017), which can regulate immunological inflammatory responses and induce lymphocyte apoptosis (Xiong et al., 2020). According to reports, TP53 is frequently mutated in UC and colorectal neoplasia that develop from UC, over 50% of colitis-induced colorectal cancer and colon neoplasia could be found with the TP53 mutation (Mäki-Nevala et al., 2021; Matsumoto et al., 2021; Arai et al., 2022). Studies have also proved that cells respond differently through TP53-mediated pathways as a result of treatment with phytochemicals from CR (Och et al., 2019; Ahmed Youness et al., 2020). HSP90AA1 is highly expressed under the stimulatory circumstances of trauma, infection, and tumors and plays an essential role in DNA damage regulation, cell cycle regulation, gene expression and carcinogenesis (Condelli et al., 2019). HSP90AA1 activity was reduced in experimental colitis, while inhibition of TREM-1 could restore its activity (Kökten et al., 2018). It has been proven that STAT3 is an effective target for inhibiting TH17 cell differentiation and attenuating colitis in IBD mouse models (Zhang et al., 2020). Mice with a STAT3 deletion in FOXP3+ Treg cells could develop aggressive colitis owing to uncontrolled TH17 responses (Chaudhry et al., 2009). In contrast, the neutralization of IL-17A alleviated chronic colitis in mice with STAT3-deficient Treg cells and decreased innate immune colitis after H. hepaticus infection (Chaudhry et al., 2009; Buonocore et al., 2010). Estrogen is an essential sex factor, and ESR1 mainly encodes the nuclear hormone receptor. Females have a more robust immune response than males in pathogen clearance and immunity boosting (vom Steeg and Klein, 2016), and this gender difference was also observed in UC (Agrawal et al., 2022). ESR1 shows the ability to suppress active inflammation and immune response (Li et al., 2022), and estradiol can suppress psoriatic inflammation in mice by regulating neutrophil and macrophage functions (Adachi et al., 2022). Moreover, methylation of ESR1 was significantly associated with UC-related colon cancer (Rosa et al., 2020). MYC, a transcription factor that regulates proliferation, differentiation, apoptosis, and epithelial-to-mesenchymal transition (Gopalakrishnan et al., 2020), is frequently overexpressed in inflammatory tissue and colitis adenocarcinomas (Pan et al., 2020). Research indicated that regulating the stability of MYC could suppress colitis-induced tumourigenesis (Raup-Konsavage et al., 2016; Parang et al., 2017). RELA is a member of the NF-κB family. The NF-κB pathways, including the RELA-dependent canonical pathway and the RELB-dependent noncanonical pathway, are essential for immune response modulation (Hoesel and Schmid, 2013). Studies at UC also found that cellular apoptosis and ROS production could also be regulated by the RELA-related signal pathway (Luo et al., 2020). In addition, phosphorylation and acetylation of RELA contribute to the transcriptional activation of various inflammatory and catabolic genes (Kleppe et al., 2018; Wang et al., 2021). What’s more, molecular docking confirmed that the key active phytochemicals of CR have ideal binding affinity with the hub targets including TP53, HSP90AA1, STAT3, ESR1, MYC, and RELA. These binding results confirmed the result that the key active phytochemicals of CR may target to TP53, HSP90AA1, STAT3, ESR1, MYC, and RELA to treat UC. It corroborated the systematic pharmacology screenings and further validated the reliability of network pharmacology in this investigation.

The GO results showed that biological processes were enriched in the regulation of inflammation, immune response, proliferation and apoptosis of cells, silencing and transcription of genes, primarily including responses to interleukin-6 and interleukin-8, T cell activation, apoptosis and proliferation of fibroblasts, and so on. KEGG pathways were further confirmed in present research, mainly including the PI3K/Akt signaling pathway, Th17 cell differentiation, inflammatory bowel disease, and so on. The PI3K/Akt signaling pathway is associated with various biological processes, including cell cycle, apoptosis, metabolism and angiogenesis (Ashrafizadeh et al., 2020). Suppression of the PI3K/Akt signaling pathway may help to regulate the balance of Tfh/Treg cells and reduce inflammatory response in experimental colitis (Jin et al., 2021; Huang et al., 2022). Further, CR and its main ingredient, Berberine, have been shown in studies to improve metabolism *via* the MAPK/PI3K/Akt signaling pathway (Cui et al., 2018; Dou et al., 2021). Th17 cell is associated with the pathophysiology of colitis, and the ratio of Th17 cells increased significantly in the splenic lymphocytes of UC patients and colitis mice (Bsat et al., 2019; Wei et al., 2021). Inhibiting Th17 cell differentiation and rebalancing Th17/Treg imbalances may be an effective treatment for chronic inflammatory disease (Qu et al., 2017). The key phytochemicals of CR, Berberine and Quercetin, can ameliorate the severity of DSS-induced colitis through regulation of Th1 and Th17 cell differentiation (Qin et al., 2010; Li et al., 2016; Yang J et al., 2022). The inflammatory bowel disease signaling pathway is a complex signaling system that includes the Toll-like receptor signaling pathway, NOD-like receptor signaling pathway and T cell receptor signaling pathways (Databases, 2022). Previous studies proved that the Toll-like receptor signaling pathway could regulated by Quercetin and Berberine (Ying et al., 2022; Zhao et al., 2022).

## Conclusion

The key phytochemicals of CR have ideal physicochemical properties and bioactivities and possess potential applications in the treatment of UC. According to previous findings and analyzed results in our research, the key molecular mechanisms of CR against UC are summarized as anti-inflammatory, immunoregulatory, anti-oxidant and anti-fibrosis by target to core genes and pathways, including TP53, HSP90AA1, STAT3, ESR1, MYC, RELA, the PI3K/Akt signaling pathway, Th17 cell differentiation and inflammatory bowel disease signal pathway. The potential and critical pharmacological mechanisms of CR against UC provide a direction for future research.

## Data Availability

The original contributions presented in the study are included in the article/ Supplementary Material, further inquiries can be directed to the corresponding authors.
