# Supplementary figures and images for: Therapeutic targets and pharmacological mechanisms of Coptidis Rhizoma against ulcerative colitis: Findings of system pharmacology and bioinformatics analysis
Source: Front Pharmacol. 2022 Nov 30;13:1037856. doi: 10.3389/fphar.2022.1037856 (PMC9748441; doi:10.3389/fphar.2022.1037856)

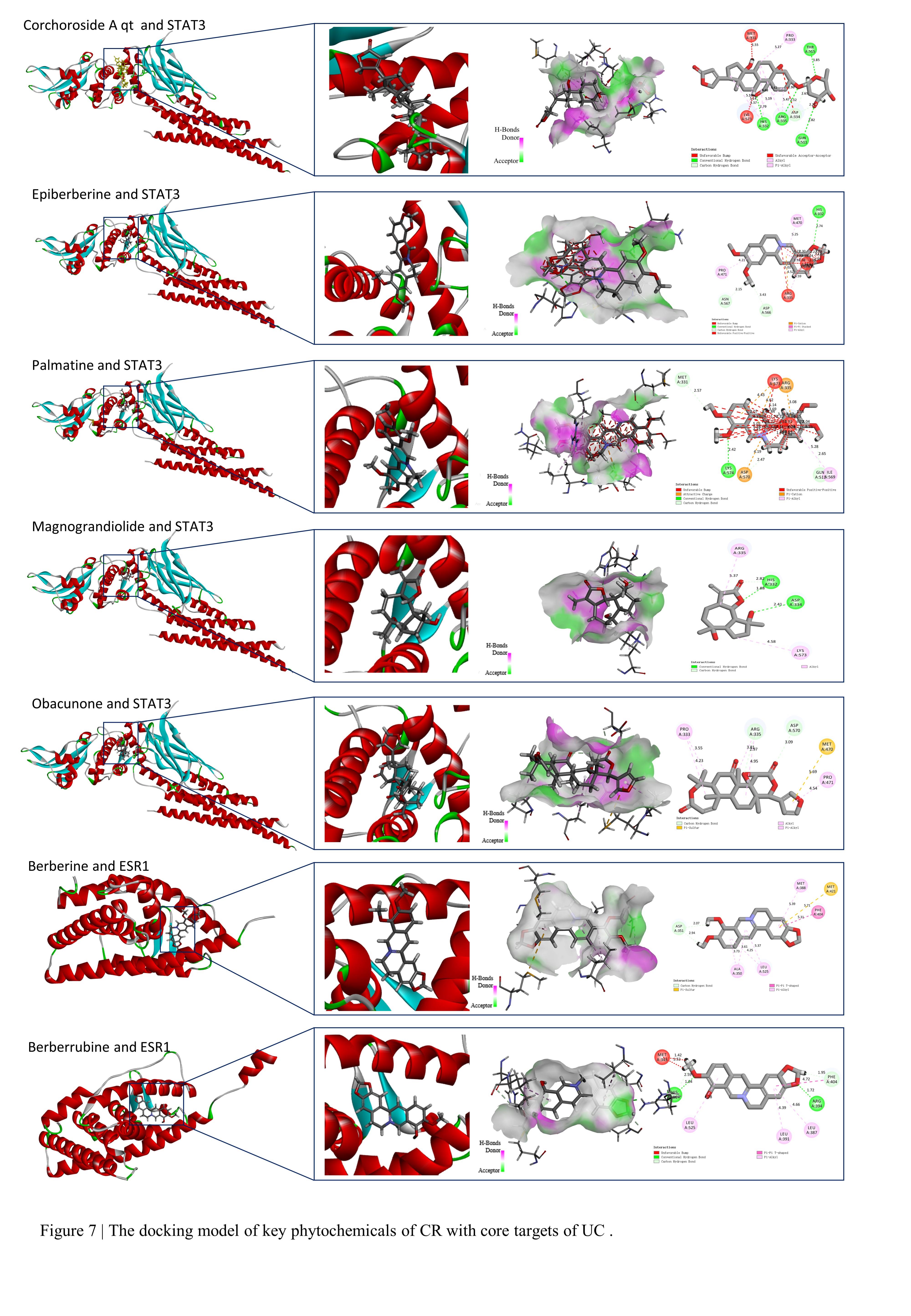

Supplement: Supplementary file 1 [file Image3.JPEG]

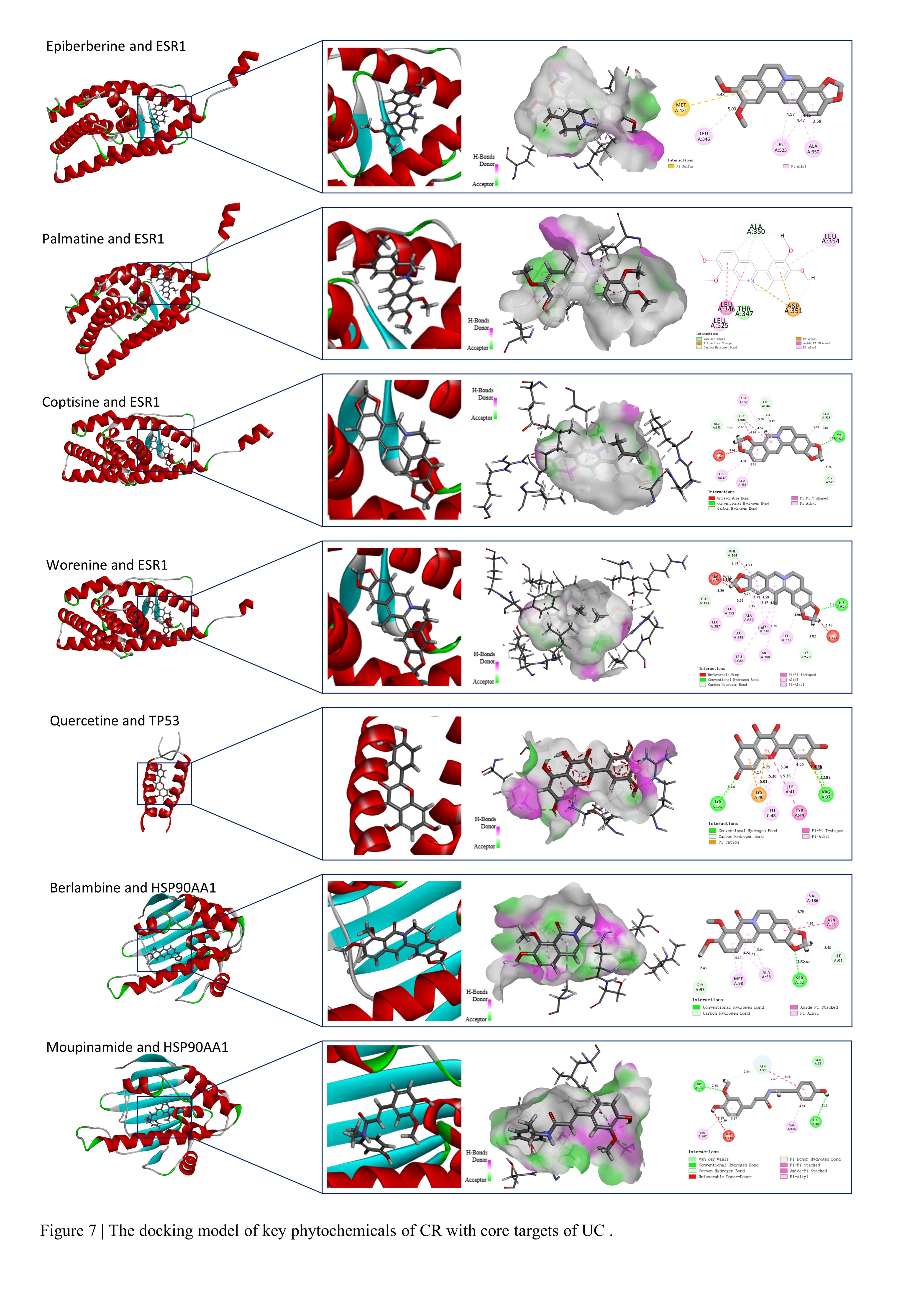

Supplement: Supplementary file 3 [file Image1.JPEG]

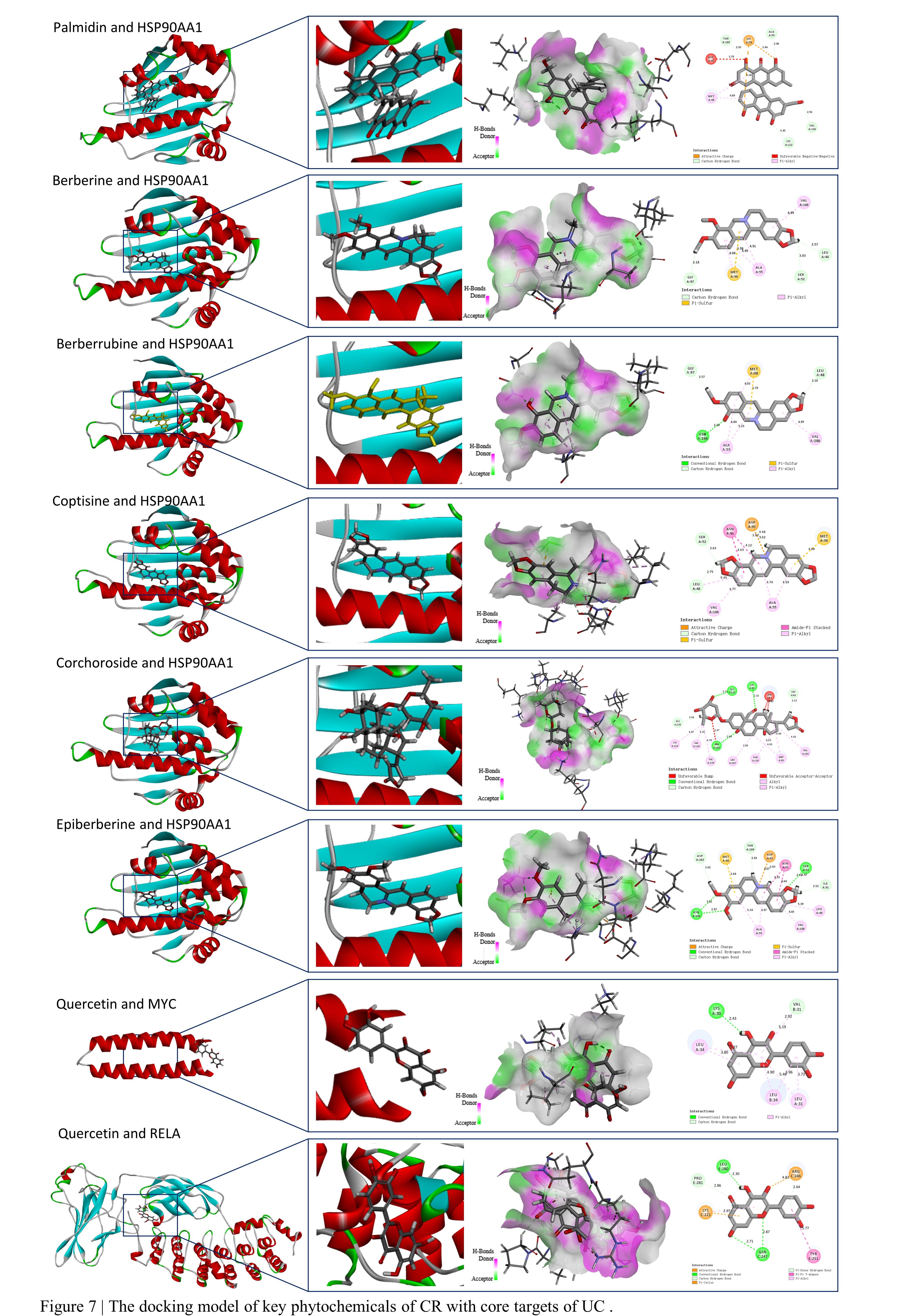

Supplement: Supplementary file 4 [file Image2.JPEG]
