# Supplementary material for: Therapeutic targets and pharmacological mechanisms of Coptidis Rhizoma against ulcerative colitis: Findings of system pharmacology and bioinformatics analysis
Source: Front Pharmacol. 2022 Nov 30;13:1037856. doi: 10.3389/fphar.2022.1037856 (PMC9748441; doi:10.3389/fphar.2022.1037856)

Supplementary figure 1. The rectangular tree diagram shows the simplify results of all the terms of GO enrichment.


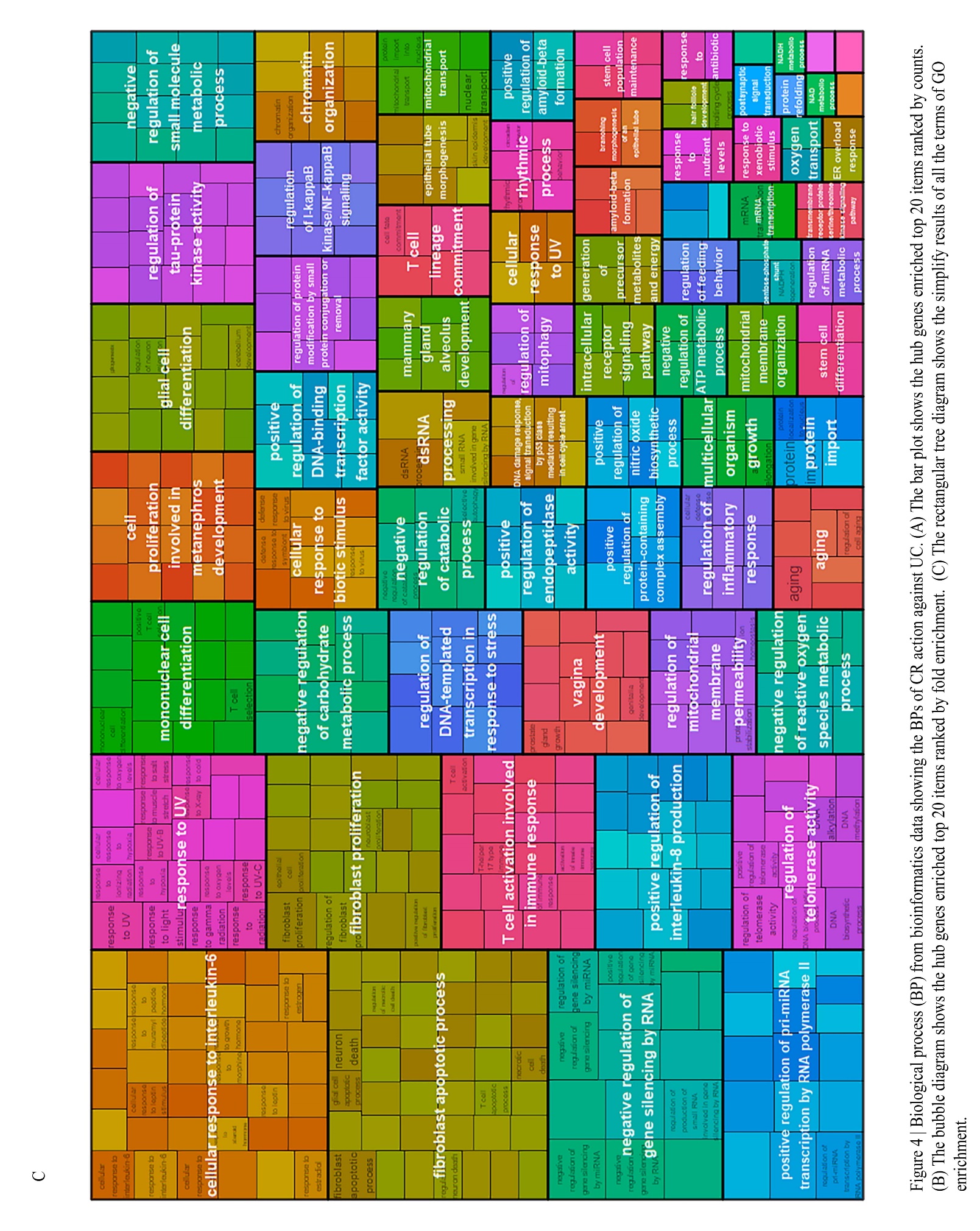


Supplementary figure 2. The schematic diagrams of binding models of key phytochemicals of CR with core targets of UC. **(A)** Palmatineand ESR1; **(B)** Worenineand ESR1; **(C)** Quercetineand TP53; **(D)** Berlambineand HSP90AA1; **(E)** Moupinamideand HSP90AA1; **(F)** Palmidinand HSP90AA1; **(G)** Berberine and HSP90AA1; **(H)** Berberrubine and HSP90AA1; **(I)** Coptisine and HSP90AA1; **(J)** Corchorosideand HSP90AA1; **(K)** Epiberberineand HSP90AA1; **(L)** Quercetin and MYC; **(M)** Quercetin and RELA.


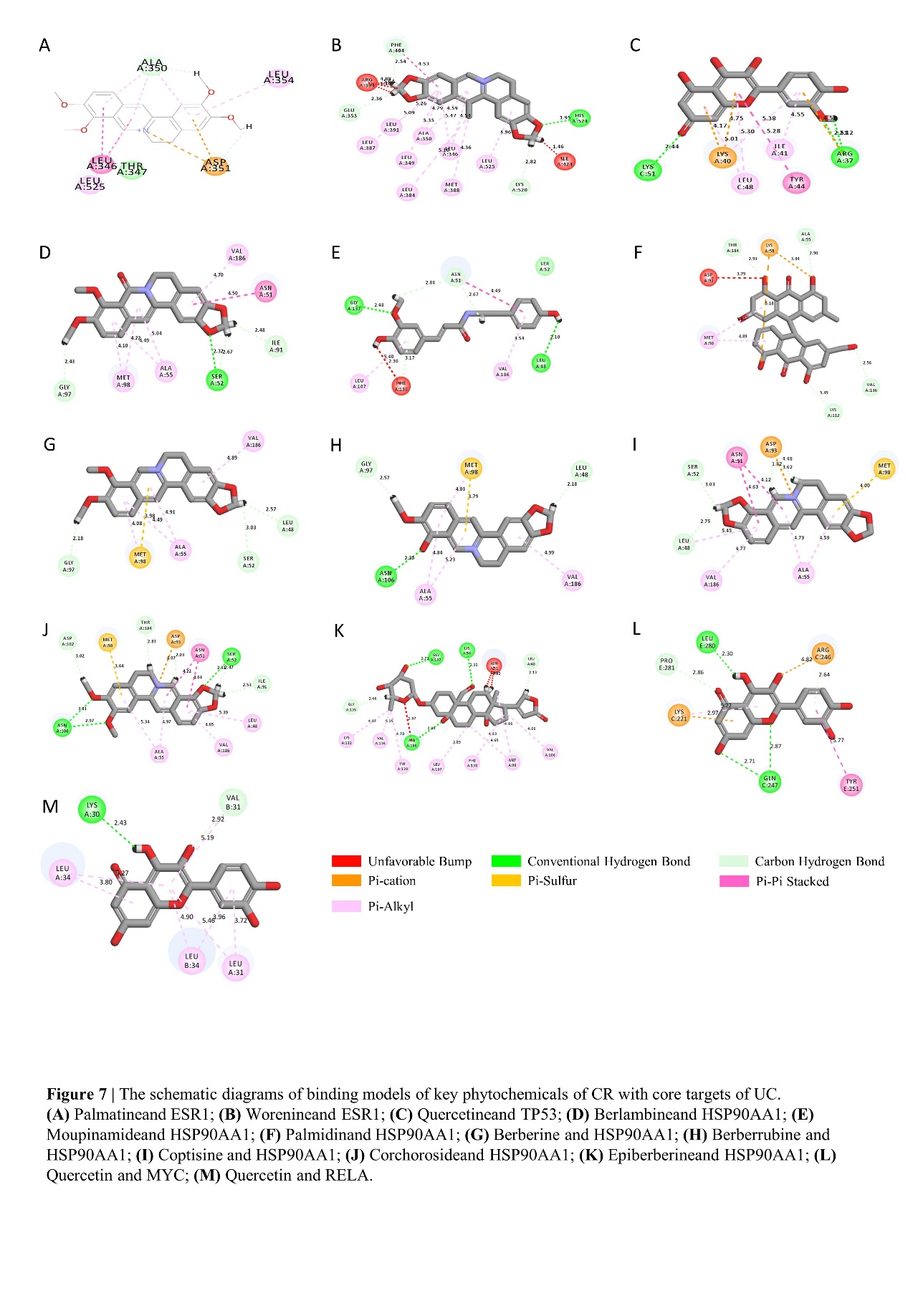

Supplement: Supplementary file 2 [file Table1.DOCX]
